# Adaptive Preheating Duration Control for Low-Power Ambient Air Quality Sensor Networks

**DOI:** 10.3390/s140305536

**Published:** 2014-03-20

**Authors:** Yoonchul Baek, Mahin K. Atiq, Hyung Seok Kim

**Affiliations:** Department of Information and Communication Engineering, Sejong University, Neungdong-ro 209, Gwangjin-gu, Seoul 143-747, Korea; E-Mails: ycbaek@sju.ac.kr (Y.B.); mahinatiq@yahoo.com (M.K.A.)

**Keywords:** air quality sensor networks, ceramic gas sensors, preheating duration, low power consumption

## Abstract

Ceramic gas sensors used for measuring ambient air quality have features suitable for practical applications such as healthcare and air quality management, but have a major drawback—large power consumption to preheat the sensor for accurate measurements. In this paper; the adaptive preheating duration control (APC) method is proposed to reduce the power consumption of ambient air quality sensor networks. APC reduces the duration of unnecessary preheating, thereby alleviating power consumption. Furthermore, the APC can allow systems to meet user requirements such as accuracy and periodicity factor when detecting the concentration of a target gas. A performance evaluation of the power consumption of gas sensors is conducted with various user requirements and factors that affect the preheating duration of the gas sensor. This shows that the power consumption of the APC is lower than that of continuous power supply methods and constant power supply/cutoff methods.

## 1. Introduction

Recently, people have moved toward spending more time indoors, such as at home or in the office. This increases the risk of exposure to various factors that are harmful to the human body. In particular, those who have a disease such as asthma, the weak, and the elderly are more affected by indoor environments, leading to health problems. To detect and clean harmful air, various healthcare devices and systems have been introduced.

A smart space can be built with healthcare devices and systems using wireless sensor networks (WSNs) utilized in various fields. Wireless body area sensor networks (WBANs) as well as WSNs can be used for sensing and transmitting the physical condition of a person or ambient information. With the help of these networks, the condition of a person can be monitored and sent to a medical team in real time, allowing the person to receive first aid in an emergency. Detecting harmful gas and preventing the human body from contacting it in advance are as important as monitoring the conditions of people. Sensors detecting harmful gas, networks, and ventilators can be used to maintain a clean environment indoors.

It is necessary to solve the problem of power consumption in WSNs and WBANs because their power sources are batteries, and frequent replacement of the battery is not feasible. To solve this problem, approaches based on low-power wireless communication, low-power processing units, node placement, and routing algorithms have been studied [1,2]. Although these existing methods can reduce the power consumption of sensor nodes, the effect is relatively small because the power consumed by the preheating of the gas sensor is large. Thus, methods considering the preheating of gas sensors for reducing the largest power consumption of the sensor networks are necessary.

Gas sensors utilize electrical properties, optic methods, acoustic methods, gas chromatography, or calorimetric methods [3]. Semiconductor and catalytic combustion gas sensors use electrical properties and calorimetric methods, respectively. Semiconductor, catalytic combustion, and solid electrolyte gas sensors are included in the category of ceramic gas sensors. For the detection of toxic gases, ceramic gas sensors are commonly used.

A semiconductor gas sensor normally utilizes a porous sintered block body consisting of polycrystalline particles of a semiconducting oxide such as SnO_2_, WO_3_, ZnO, or In_2_O_3_ [4]. This sensor has been widely used in practical applications because it can detect various target gases with a fast response time and low cost. However, a commonly used n-type semiconductor gas sensor has serious drawbacks in terms of high power consumption because of the need to preheat the sensor to reach between 300 °C and 400 °C to sense the target gas accurately. The operating principle of the catalytic combustion gas sensor is similar to that of a semiconductor gas sensor. A catalytic combustion gas sensor is commonly used to detect inflammable gases. Heating a platinum wire in a ceramic bead consumes significant power because the catalyst layer should reach temperatures higher than 500 °C [5,6] for accurate detection. A solid electrolyte gas sensor uses ionic conductivity by moving ions instead of electronic conductivity. This sensor has outstanding accuracy in detecting carbon dioxide but requires preheating the gas sensor to reach a temperature of 400 °C to 500 °C for accurate detection [7].

A ceramic gas sensor should be preheated to a certain temperature for accurate detection of the reacting gas and steady operation of the gas sensor. In an energy-constrained WSN, it is difficult to keep the sensor heated continuously because excessive energy is wasted. Several solutions for reducing power consumption have been proposed. To decrease the high power consumption in a gas sensor, the use of a microheater in the membrane of a gas sensor was proposed in [8], and a new fabrication method for a membrane was proposed in [9]. The required power differs depending on the material used in the microheater. The microheater forces the temperature to increase by more than 11% by using titanium nitride rather than platinum in [10]. In [11], the ideal size of a microheater was proposed for low power consumption. The proposed coplanar microheater fabrication method presented in [12,13] can reduce the fabrication cost and stages of the sensor that uses a microheater. However, fabrication of the sensors is time consuming and requires significant cost. Therefore, a feasible low-cost method for reducing the power consumption during the preheating of the gas sensors is required.

In this paper, we propose a novel approach, called adaptive preheating duration control (APC), for solving the power consumption problem incurred by preheating the gas sensor to facilitate accurate detection. As far as authors know, there is no other method considering the control of preheating duration for saving energy consumption of gas sensor systems. In APC, power is supplied to the sensor until a sensing value is obtained that fulfills the requirements of the user; power is removed once sensing is finished. The APC uses the characteristics of preheating to reduce the power consumption of the gas sensor.

The continuous power supply method (CPSM) continuously supplies the sensor with power. In the constant power supply/cutoff method (CPSCM), the sensor system uses the same pattern of preheating and power removal repetitively that has the preheating duration required for the initial state of the sensor. The temperature pulsed operation method (TPOM) regulates the magnitude of power according to the sensor temperature [14], Table 1 lists different features of CPSM, CPSCM, TPOM, and APC.

The remainder of the paper is organized as follows: Section 2 presents the proposed APC method in detail. The performance of APC is evaluated in Section 3 in terms of its power consumption and error rate. Finally, the concluding remarks are provided in Section 4.

## 2. Adaptive Preheating Duration Control Method

**Figure 1.**
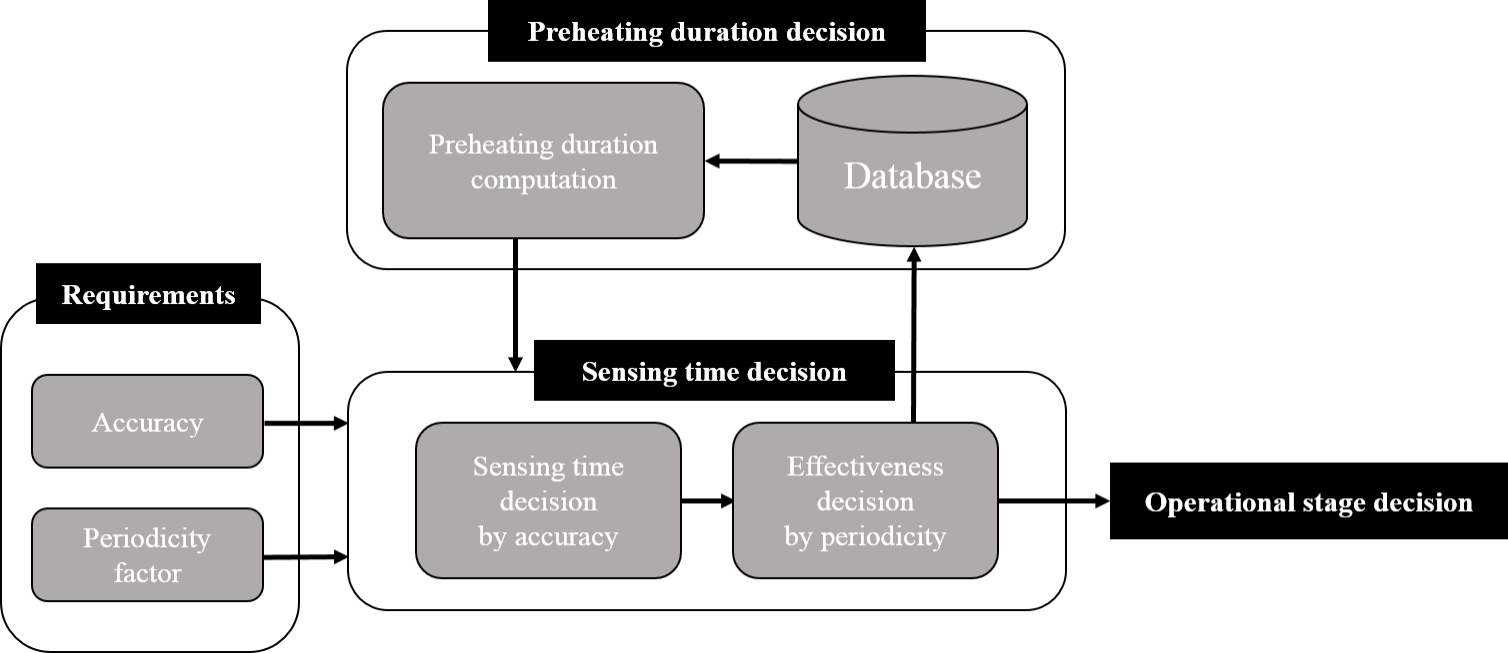
Overall block diagram of the APC method.

As depicted in Figure 1, the APC method is organized into requirements, the preheating duration decision, the sensing time decision, and the operational stage decision. In APC, the sensor measures gas concentration while preheating, and at the sensing time, it stops heating and stores the sensor output as a final sensing value. The sensing time is chosen by comparing the gradient in the time-variant graph of sensor outputs, with the gradient determined by the accuracy requirement, and then the effectiveness of the determined sensing time is determined considering the periodicity factor requirement.

A sensor-actuator network consisting of air quality sensors and ventilators needs to make the operation of the actuator suitable for the sensing value, considering the measurement error range of the sensor. Thus, the network uses the accuracy as one of the criteria with a periodicity factor. The periodicity factor is based on the difference between the sensing time determined via accuracy criteria and the required periodic transmission start time. The periodicity factor is used to confirm whether the time when determining the sensing value, *i.e.*, the sensing time, follows the given period of sensing time. The accuracy and periodicity factor are required for the APC and can determine the sensing time within the preheating duration, *i.e.*, the time during which the power supply to the sensor is on. The preheating duration could be calculated by factors saved in the database, such as the preheating duration required for the initial state, transmission period, correlation coefficient, accumulated power supply, and cutoff duration in the sensor. The correlation coefficient is defined as the ratio of the preheating duration to the time required to return to the initial state after preheating. The accumulated power supply and cutoff duration are saved in the database and updated after the sensing time is determined.

The APC obtains the required result before the completion of preheating and can help reduce power consumption. The APC disconnects the power supply from the sensor immediately after sensing. The sensor network determines the necessary preheating duration from the previous heating/cooling durations and then preheats the sensor for that time. By executing this process repeatedly, the APC can increase the efficiency of the power consumption.

### 2.1. Requirements

In general, users consider the accuracy of the sensor and select a sensor satisfying their requirements. The APC has the gas sensor measure gas concentration before the sensor preheating is finished. Thus, the error in the sensing result when using APC may be more than that in the sensing result obtained after the sensor preheating is completed. The measurement error will increase if the power supply time is excessively reduced to save power consumption. Thus, the accuracy is set considering the user requirement for meeting the desirable error tolerance level. The sensor networks cannot operate in a stable state if they use meaningless values or erroneous sensor measures. Thus, accuracy should be used as a primary requirement for making sensing time decisions in the APC method. The accuracy requirement of the user is used not only to determine the sensing time but also to control the operational stage such as the alarm system in a smart space.

The second requirement is the periodicity factor, which determines whether the sensing time is matched with the transmission start time of the WSN. The periodicity factor (PF) indicates the allowable time difference between the sensing time and transmission start time and is calculated as follows:

(1) $$\text{PF}=\frac{T-S}{P}\times 100$$

where T, S, and P denote the transmission start time, sensing time, and period, respectively. A periodicity factor of 0% means that the sensing time and the transmission start time are equivalent, and the sensor network should measure the target gas at the predetermined transmission start time. When the specific sensor value is transmitted periodically, if there is a large time difference between the sensing time and transmission start time, then this large variation will cause the measured value to be outdated.

Thus, users have to consider the time-variant concentration and harmfulness of the target gas when setting the periodicity factor. For instance, when sensing the carbon dioxide in the atmosphere, the periodicity factor can be set to a high value because the harmfulness of carbon dioxide to the human body is less than that of carbon monoxide.

### 2.2. Preheating Duration Decision

**Figure 2.**
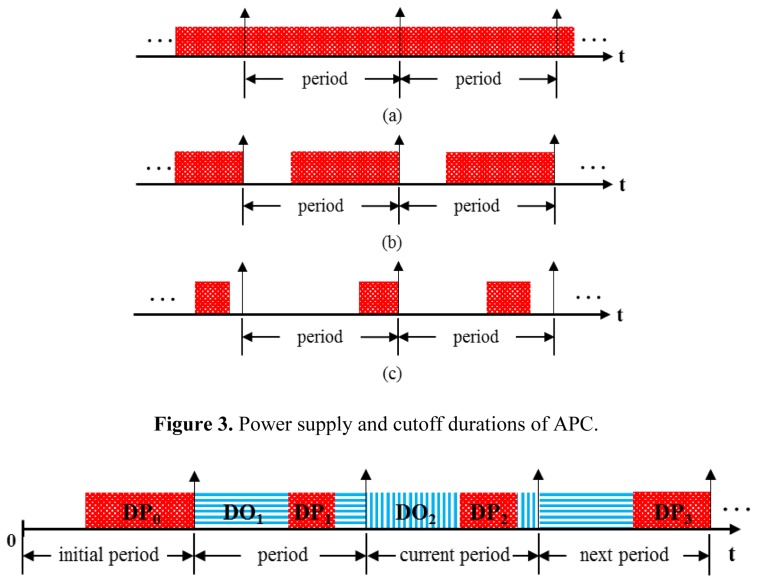
Timing diagrams of power supply methods for preheating sensors. (**a**) Continuous power supply method; (**b**) Constant power supply/cutoff method; (**c**) Power supply and cutoff by APC method.

The gas sensor networks generally use the continuous power supply method shown in Figure 2a. Using that method in applications for intermittent data sensing leads to power wastage because the duration of the supplied power is longer than the required power supply time. This problem becomes more severe when the transmission period is gradually increased. One of the methods for solving this problem is shown in Figure 2b and uses the constant power supply duration and cutoff duration over all periods repeatedly. The power is supplied only during the required preheating duration for normal sensor measurement, and then the power supply is paused immediately after sensing. The blocks shown in Figure 2a–c denote the preheating durations until the power supply is cut off. The empty part denotes the power cutoff periods. The method in Figure 2b consumes less power for preheating the sensor than the method in Figure 2a because of the cutoff duration. The preheating duration indicated in the specification of the sensor is the power supply time required in the initial state. Once the sensor is heated, the necessary preheating duration after pausing the power supply becomes shorter. Thus, to reduce the power, the preheating duration should be changed based on the effect of the previous preheating duration. The method in Figure 2b has an unnecessary power consumption duration because it uses a constant preheating duration throughout the operation of the sensor. In addition, if the transmission period is equal to or shorter than the preheating duration required for the initial state, Figure 2b becomes equivalent to Figure 2a. Figure 2c shows an example of the APC method in which the preheating duration can be decreased, considering the previous preheating state. For these reasons, the APC method can reduce the power consumption of gas sensor networks.

**Figure 3.**
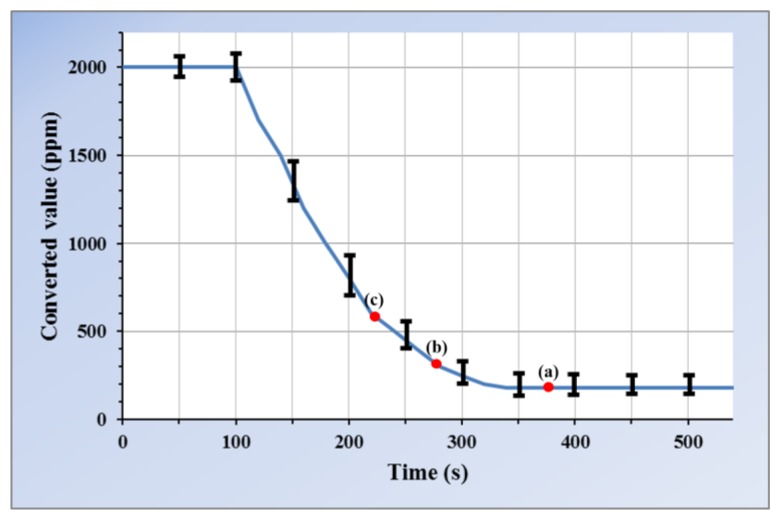
Power supply and cutoff durations of APC.

To apply the APC method, the sensor network should know the preheating duration required for the next period. The preheating duration DP is calculated as the product of a preheating coefficient and the initial preheating duration, which indicates the preheating duration required at the initial sensor state. The preheating coefficient is adaptively adjusted by referring to the accumulated power supply duration, cutoff duration, and correlation coefficient. The accumulated power supply duration is calculated by summing up the power supply durations from the initial period to the current period, and the accumulated cutoff duration is calculated by summing up the cutoff durations up to the current period and the duration excluding the preheating duration in the next period, as shown in Figure 3.

The required preheating duration in the next period is calculated as

(2) $$DP_{i+1}=\frac{\sum_{n=0}^{i}\left(DO_{n})+(P-DP_{i+1}\right)}{\sum_{n=0}^{i}(DP_{n})\times C}\times DP_{0}(DO_{0}=0,i\geq 0)$$

where DO and C are the power cutoff duration and correlation coefficient, respectively. C is affected by the fabrication stage of the sensor, and the temperature and humidity of the measurement environment. Subscript *i* is used to indicate the sequence of the period. DP_0_ is the initial preheating duration, which is determined by the fabrication step of the sensor. DO_0_ is set to zero because it does not affect the next preheating duration. The correlation coefficient C is the ratio of the preheating duration to the time required to return to the initial state after preheating. For instance, if a specific gas sensor requires 10 min for preheating in the initial state and requires a power cutoff duration of 120 min to return to the initial state; then C is 12. In addition, when the power cutoff duration lasts more than 120 min, the sensor state assumes the initial state. Thus, although the power cutoff duration lasts longer than 120 min, the preheating coefficient is set to one to prevent the next preheating duration from being higher than the initial preheating duration in Equation (2).

By transposition of DP_i+1_ to the left-hand side of Equation (2), we obtain:

(3) $$\left(1+\frac{DP_{0}}{\sum_{n=0}^{i}(DP_{n})\times C}\right)\times DP_{i+1}=\frac{\{\sum_{n=0}^{i}(DO_{n})+P\}\times DP_{0}}{\left\{\sum_{n=0}^{i}(DP_{n})\times C\right\}}(DO_{0}=0,i\geq 0)$$

and then dividing both sides by $\left(1+{\text{DP}}_{0}/(\sum_{\text{n}=0}^{\text{i}}\left({\text{DP}}_{\text{n}}\right)\times \text{C})\right)$produces:

(4) $$DP_{i+1}=\frac{DP_{0}\times \{\sum_{n=0}^{i}\left(DO_{n})+P\right\}}{DP_{0}+\{\sum_{n=0}^{i}\left(DP_{n})\times C\right\}}(DO_{0}=0,i\geq 0)$$

Preheating starts at the time obtained by Equation (4) before the next transmission period.

**Figure 4.**
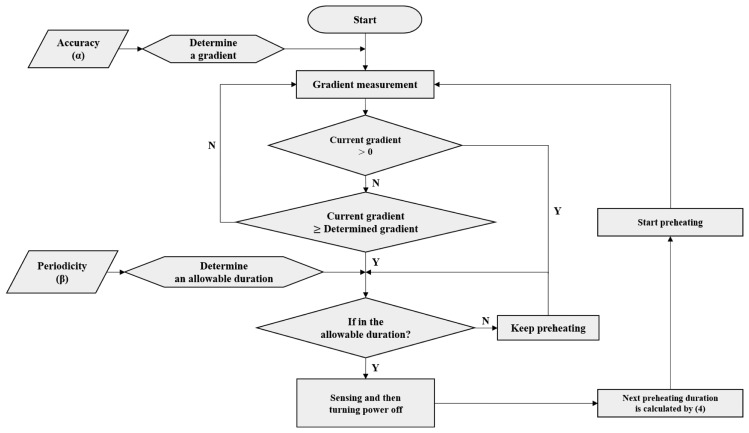
Example of preheating duration.

A sensor measures within the preheating duration, and its sensing result is obtained by converting the output voltage of the sensor to the concentration of the target gas. For instance, the GHFS41-P110 solid electrolyte gas sensor [15] measures the concentration of carbon dioxide using:

(5) $$\text{C}=10^{\frac{9.410-V}{2.705}}[\text{ppm}]$$

where V denotes the output voltage, and C is the carbon dioxide concentration, which has a range between 200 and 2,000 ppm. Figure 4 shows the change in carbon dioxide concentration for the preheating duration of the GHFS41-P110. The concentration is saturated at 200 ppm after heating the sensor. The output voltage of the sensor is varied within a standard deviation by the measurement environment except for the concentration of the target gas. In addition, the maximum and minimum values and saturation time are varied on the basis of the inherent properties of each sensor.

The properties of the preheating duration are changed under various conditions. The preheating duration differs depending on the concentration of the target gas in the measurement environment. For example, if the concentration is 1,000 ppm, the sensor output decreases gradually from 2,000 ppm and is saturated near 1,000 ppm. In this way, if the sensor output variance is maintained within a standard deviation during a certain time, it is determined to be equal to the target gas concentration within the error range. The power supply to the sensor is removed promptly after confirming the periodicity factor, which is one of the user requirements. After saturation, the sensor output is determined for a certain time, which is performed differently given the required accuracy and power consumption.

### 2.3. Sensing Time Decision

**Figure 5.**
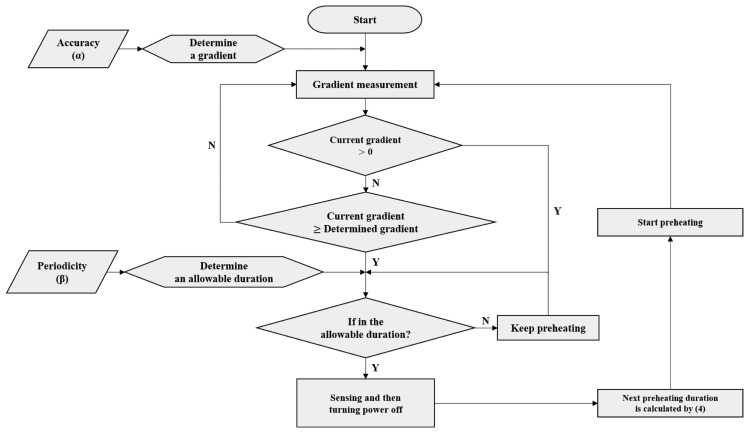
Flow of sensing time decision.

The sensing time is the point in time for the sensor system to determine the concentration of the target gas within the preheating duration and is determined in accordance with user requirements. Figure 5 shows the determination process of the sensing time. In the first stage, the WSN receives the accuracy and periodicity factor requirements of the user. The accuracy is denoted by α. The accuracy of the sensor becomes 90% if the error in the range of the sensor is 10%. The decision criterion for the sensing time required in the preheating duration is based on the accuracy and periodicity factor set by the user. In this paper, the APC method uses the gradient of the sensor output, which can incorporate the variations in sensing values as the decision criterion. If the accuracy is set to the maximum value, the gradient that determines the sensor saturation becomes zero. The maximum accuracy uses the gradient that maintains the specific value in the preheating duration, e.g., point (a) in Figure 4. If a high level of accuracy is not required, the gradient can be set such that the measurement can be sensed more quickly, e.g., points (b) and (c) in Figure 4. If the sensing time is determined, target sensing is conducted with decreased accuracy in the measurement. This is because the value is obtained before sensor saturation. Thus, accuracy α, as one of user requirements, determines the gradient of the sensor output as the primary decision criterion for the sensing time.

Once the sensor preheating starts, the gradient of the sensor output is measured every predetermined interval. The sensor output at the starting point of preheating is also varied when repeating the power supply and cutoff. For example, at the initial state, the GHFS41-P110 sensor output is decreased gradually from 2,000 ppm. However, if the cutoff duration is not sufficient to return the sensor to the initial state before preheating, the sensor output in the starting point of preheating becomes lower than 2,000 ppm. This is because the sensor cannot return to the initial state because of the remaining impact of the previous period of preheating. If the sensor output at the starting point of preheating is lower than the concentration of the target gas, it does not decrease as in Figure 4, and the gradient attains a positive value. In this case, the sensor output changes rapidly from the initial value to the saturation value of the sensor, and then the sensor network determines whether the sensing time is within the duration satisfying the periodicity factor requirement. On the other hand, if the gradient is equal to or lower than zero, the sensor network checks whether the current gradient is equal to or higher than the gradient that meets the accuracy requirement. If the current gradient is equal to or higher than this value, the sensor network determines that the sensing value satisfies the required accuracy.

If the sensing value is determined by the accuracy, the sensor network needs to consider whether the current sensing time is within the allowable duration. The allowable duration is obtained as the product of the periodicity factor and the transmission period. The allowable duration becomes the second criterion in the sensing time decision. For instance, if the periodicity factor is 10%, and the transmission period is 30 s, the allowable duration becomes 3 s. The sensing time can be chosen to be more than 3 s before the next transmission start time. Therefore, the sensing value is not saved even if it satisfies the required accuracy during the time from 30 to 57 s. In case that the sensing time is not within the allowable duration, the determined sensing value is invalid. Thus, the sensing value is obtained when the accuracy and periodicity factor are valid. The power is removed immediately after the obtained sensing value is saved in the memory. The sensing value is transmitted at the transmission start time, which is 60 s. After sensing, the next preheating duration is calculated using the cumulative power supply and cutoff durations. This procedure is repeated every transmission period.

### 2.4. Operational Stage Decision

**Figure 6.**
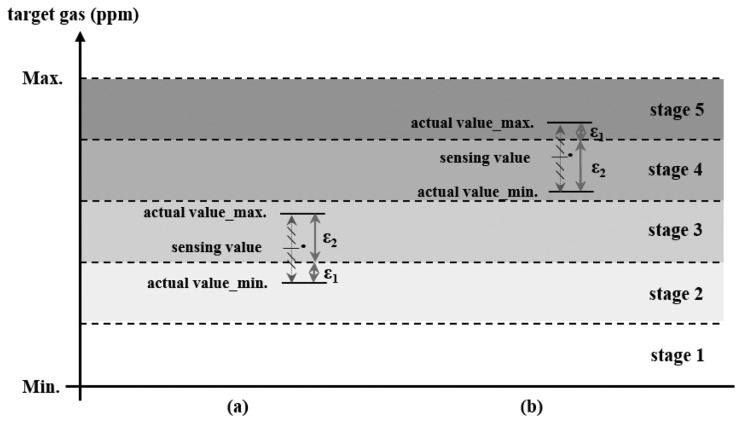
Examples of (**a**) a higher operational stage decision and (**b**) a lower operational stage decision.

A ceramic gas sensor can be used in various fields for different purposes, e.g., healthcare systems, alarms for toxic gas discharge, and ambient air quality sensor networks. When used for monitoring, the sensor network may transmit the sensing value with or without the error range. If the sensor network uses this value including errors to control the operational stages, the accuracy of the stage decision may be lower than that of the requirement. If the error for the operational stage decision meets the user requirement for accuracy, the sensor network can work well. Otherwise, it may incur problems such as unnecessary power consumption from the ambient air quality control equipment or exposure to toxic gas resulting from errors of stage determination. The power of the equipment is wasted in the sensor network if the determined stage is higher than the required stage because the equipment consumes significantly more power than the sensor. If a lower stage is determined, it can cause air quality to exceed the limit of the environmental standards and may cause damage to the human body. Figure 6a,b show cases involving power waste and an increase in exposure time to toxic gases.

In Figure 6a,b, the sensing values are marked with “ · ”in the range of actual value_min to actual value_max, which indicates the error range of sensing. The operational stage required by the actual concentration value can differ from that determined by the sensing value, as shown in Figure 6. Let ε_1_ denote the error occurrence interval, and the error rate of the operational stage decision can be calculated as the ratio of ε_1_ to ε_2_. A state decision error occurs because of the difference between the operational stage determined by the sensing value and the operational stage required by the actual concentration. Thus, the user has to set the accuracy such that the error rate should not exceed the allowable error rate. The allowable error rate is calculated as 100 − α (%), where α is the accuracy as a user requirement. If the stage decision error rate is higher than 100 − α (%), a solution is required for a reduction of both the unnecessary power consumption of equipment and the exposure time of the toxic gases.

The abovementioned issue can be solved as follows: using more accurate sensors, widening the interval of the operational stage by reducing the number of operational stages, and lowering the accuracy requirement. However, these methods cost more or degrade performance. In the proposed solution for addressing the problem, the ambient air quality sensor network adjusts the operational stage by using:

(6) $$\frac{\varepsilon_{1}}{\varepsilon_{1}+\varepsilon_{2}}\times 100>100-\alpha$$

If Equation (6) is true, the ambient air quality sensor network changes the operational stage. Otherwise, it proceeds through the stage including the sensing value. For instance, in Figure 6a, the sensor network only has to operate at stage 2 when there is an excessive error rate, which can prevent unnecessary power consumption. In this way, in Figure 6b, the exposure time to toxic gases can be reduced.

## 3. Performance Evaluation

In this section, the power consumption of sensors using the APC method is compared with that of other methods under various conditions. The compared methods are the continuous power supply method and constant power supply/cutoff method, as shown in Figure 2a,b.

For the performance evaluation, parameters of a solid electrolyte gas sensor (GHFS41-P110) measuring the concentration of carbon dioxide are used. The sensor is capable of measuring in the range of 200 to 2,000 ppm and has an accuracy of 90%, which is the maximum measurable accuracy of the sensor. The preheating duration of the sensor is 9 min in the case of a 4-W power supply. Other features of the sensor for the performance evaluation are obtained through the experiments. The basic conditions of the experimental environment used to analyze the characteristics of the sensor are listed in Table 2.

The power consumption of the APC was evaluated, changing the requirements such as the accuracy and periodicity factor. In addition, the APC was compared with other methods under variation of the transmission period, concentration of target gas, and amount of power supply, which affect the preheating duration of the sensor.

**Figure 7.**
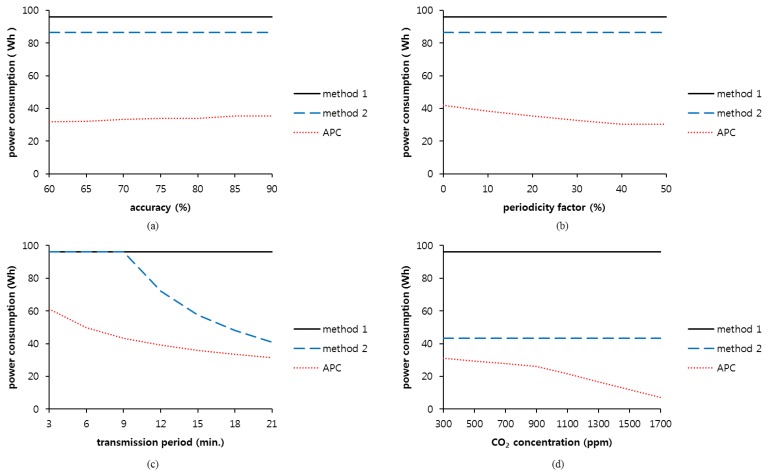
Comparison of the power consumption *vs.* (**a**) accuracy; (**b**) periodicity factor; (**c**) transmission period and (**d**) concentration of carbon dioxide.

Figure 7 shows the amount of power consumed by the sensor consume for different accuracy requirements, periodicity factors, transmission periods, and carbon dioxide concentrations. The continuous power supply method and constant power supply/cutoff method are termed method 1 and method 2, respectively. To minimize the impact of accuracy on the power consumption, a 50% periodicity factor is used in Figure 7a. The transmission period is set to 10 min, and the concentration of carbon dioxide gas in the experimental environment is set to 600 ppm.

When comparing the power consumption over 24 h, method 1 consumes 96 Wh, and method 2 consumes 86.4 Wh. However, in the APC method, power consumption is relatively small—in the range of 31.78 Wh with 60% accuracy to 35.50 Wh with 90% accuracy. There is a difference of 3.72 Wh between the maximum and minimum power consumptions. More power is consumed as accuracy increases. At the 60% accuracy requirement, the sensing time is less than at the 90% accuracy requirement by 56 min. The power consumption can be reduced by 64.92% and 61.03% when using the APC method as compared to methods 1 and 2, respectively.

Figure 7b shows the impact of the periodicity factor on the power consumption for three different methods. The accuracy is maintained at the maximum value of 90% to minimize the effect of accuracy on the sensing time under equivalent conditions. The results of methods 1 and 2 show power consumptions of 96 Wh and 86.4 Wh, respectively, which are equivalent to the values in the simulations at that accuracy. This implies that methods 1 and 2 are not affected by the periodicity factor. However, when using APC, the power consumption decreases at a higher periodicity factor because it can determine the sensing time flexibly based on the periodicity factor.

In this simulation, the power supply duration of method 1 is 10 min between transmission periods because the transmission period is set to 10 min. The duration of method 2 is 9 min. However, the preheating duration of the APC is in the range of 9 min or less in consideration of the previous preheated state of the sensor.

Figure 7c shows the impact of the transmission period on the power consumption of the sensor. The accuracy and periodicity factors are set to 90% and 0%, respectively, to observe the impact of the transmission period on the power consumption. Method 1 consumes 96 Wh for 24 h because of its continuous power supply, regardless of the transmission period. However, method 2 supplies the power for 9 min before every transmission period and removes the power after sensing. Method 2 consumes 96 Wh, which is equivalent to the power consumption of method 1 when the transmission period is shorter than the preheating duration required in the initial state. At a transmission period of more than 9 min, the power consumed for 24 h is decreased because of a gradual increase in the blocked duration of the power supply. The consumed power for the APC method also gradually decreases as the transmission period becomes longer.

Figure 7d shows the impact of carbon dioxide concentration on power consumption. In methods 1 and 2, the power consumption is not affected by the concentration of carbon. However, the APC method decreases the power consumption as the concentration of carbon dioxide increases. Higher concentration results in earlier saturation, as shown in Figure 4. The sensing time also occurs earlier, and the power cutoff duration increases. The periodicity factor is set to 50%.

**Figure 8.**
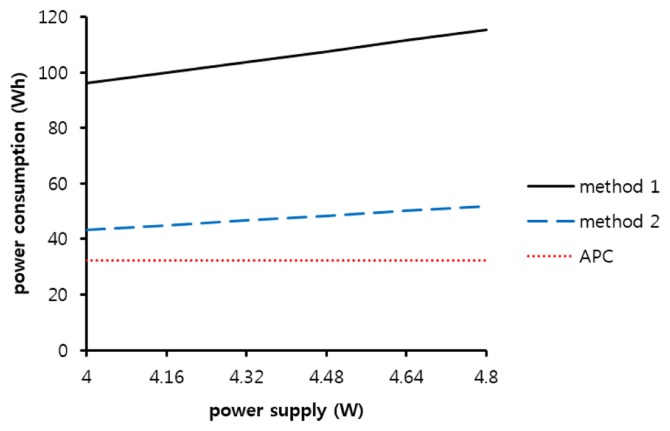
Power supply *vs.* power consumption.

In previous simulations, the power supply was set to a constant value of 4 Wh. However, the GHFS41-P110 sensor can be supplied with a power up to 4.8 Wh instead of 4 Wh because the input voltage is available in the range of 5 to 6 V with a 0.8-A current. Figure 8 shows the power consumed by the sensor with the increased power supply. The supplied power is calculated as the product of the magnitude and supply time of the power. In methods 1 and 2, the power supply durations are constant for every transmission period, and the magnitude is increased, so the amount of power consumption is gradually increased. The power consumption of method 1 increases from 96 Wh, and the simulation result of method 2 increases from 43.2 Wh. However, the power consumption of APC is constant because the preheating duration of the sensor is adaptively reduced.

If the range of the allowable power supply to the sensor is large, methods 1 and 2 may encounter a serious problem in which the power consumption gradually increases in proportion to the supplied power. However, the APC is able to maintain its power consumption.

**Figure 9.**
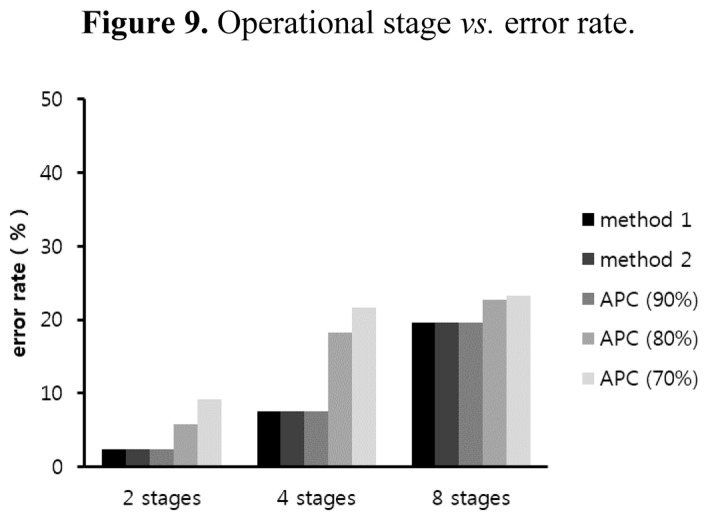
Operational stage *vs.* error rate.

In the previous accuracy simulation result, the power consumption is decreased when the accuracy is set to a low value because the sensing time becomes earlier. The accuracy requirement can determine the operational stage as well as the sensing time. We measure the error rate of the operational stage under the accuracy requirement. Figure 9 shows the error rate when varying the accuracy requirement and the number of operational stages. This simulation was conducted for methods 1 and 2 with 90% accuracy and method 3 with 70%, 80%, and 90% accuracy. All methods were evaluated for 150 h with a transmission period of 9 min. The concentration of carbon dioxide in the measurement environment was changed from 200 to 2,000 ppm, following the range of the GHFS41-P110 sensor specification. The operational stages can be divided into two, four, and eight stages. For instance, the two-stage system only has ON and OFF states in that equipment such as the alarm runs when the parameter is higher than a threshold and pauses otherwise.

APC with 90% accuracy has the same error rate for the operational stage as methods 1 and 2. Thus, for instance, if the sensing value is 1,000 ppm, the actual concentration of carbon dioxide can be expected to range from 909 to 1,111 ppm. The ambient air quality sensor network assumes the OFF state if the sensing value is lower than 1,100 ppm, and otherwise, the equipment assumes the ON state. In this example, the sensor network is set to the OFF state because the sensing value is 1,000 ppm. However, because the required operational state is ON when exceeding 1,100 ppm, in this case, an error occurs.

The minimum and maximum errors in the two-stage system are 2.44%, and 9.17%, respectively. In this condition, the sensor network can operate without any problem because the error rate is lower than the allowable error rate. The four- and eight-stage systems are divided into increments of 450 ppm and 225 ppm, respectively, from 200 to 2,000 ppm. In eight-stage systems, the error rates are 19.61% with 90% accuracy and 22.69% with 80% accuracy. In only these cases, the allowable error rates are exceeded by 9.61% and 2.69%, respectively. Therefore, we need to reduce the error rates by using more accurate sensors, widening the interval of the operational stage, or lowering the accuracy requirement. However, the proposed method in Section 2.4, which adjusts the operational stage using a simple algorithm, can maintain the sensor network without any problem, additional cost, or degradation of performance.

The highest error rate occurs with a probability of 23.28% when the accuracy is set to 70% in the eight-stage system. This error rate is relatively high, but it is included in the range of the allowable error rates at 30%. If a specific system approves the high error rate, it can quickly determine the sensing time and reduce power consumption. Thus, we need to set the requirements properly, considering features of the measurement environment.

## 4. Conclusions

In this paper we have proposed an APC method that considers accuracy and periodicity as user requirements and reduces the power consumption of air quality gas sensor networks. Considering user requirements, the proposed method reduces the duration of the preheating necessary for making accurate measurements by turning the power supply to the sensor on and off. The power consumption of the APC was compared with the power consumption of a continuous power supply and constant power supply/cutoff methods. The performance evaluation was conducted under conditions with varying accuracies, periodicity factors, transmission periods, concentrations of target gas, and amounts of supplied power. The simulation results show that the power consumption of the APC is lower than that of other methods. The APC is able to solve the problem of high power consumption for systems related to ambient air quality sensor networks, which are always power constrained and cannot afford frequent battery changes, by reducing unnecessary power consumption from the preheating duration of the gas sensor.

## Figures and Tables

**Table 1.** Features of preheating control methods for gas sensors.

	**CPSM**	**CPSCM**	**TPOM**	**APC**
**Power supply pattern**	Continuous supply	Constant supply/cutoff	Magnitude regulation	Adaptive supply/cutoff
**Error control function**	Not used	Not used	Not used	Used
**Availability for conventional sensors system**	Yes/Software change	Yes/Software change	No/Hardware change	Yes/Software change

**Table 2.** Experimental environment.

**Parameter**	**Value**
Temperature	20°C ± 5°C
Humidity	RH 65% ± 10%
Pressure	1 atm
